# Draft Genome Sequence of Enterotoxigenic *Escherichia coli* Strain W25K

**DOI:** 10.1128/genomeA.00593-14

**Published:** 2014-06-26

**Authors:** Wenkai Ren, Gang Liu, Jie Yin, Shuai Chen, Tiejun Li, Xiangfeng Kong, Yuanyi Peng, Yulong Yin, Philip R. Hardwidge

**Affiliations:** a Laboratory of Animal Nutrition and Health and Key Laboratory of Agro-Ecology, Institute of Subtropical Agriculture, the Chinese Academy of Sciences, Changsha, Hunan, People’s Republic of China; b Chongqing Key Laboratory of Forage and Herbivore, College of Animal Science and Technology, Southwest University, Chongqing, China; c Department of Diagnostic Medicine/Pathobiology, Kansas State University, Manhattan, Kansas, USA

## Abstract

Enterotoxigenic *Escherichia coli* (ETEC) is a major cause of diarrheal disease in humans and newly weaned pigs. Here, we report the draft genome sequence of ETEC strain W25K, which causes diarrhea in piglets.

## GENOME ANNOUNCEMENT

Enterotoxigenic *Escherichia coli* (ETEC) causes travelers’ diarrhea and is a leading cause of infectious diarrhea in children from developing nations (1). ETEC is also a major cause of diarrhea in newly weaned pigs (2). The K88 (F4) fimbrial adhesin and heat-stable (ST) and heat-labile (LT) enterotoxins have been identified as important virulence factors leading to diarrheal diseases in piglets (3, 4). Although the complete genomes of three ETEC strains that infect humans, *E. coli* H10407 (5), E24377A (6), and B2C (7), have been published, no ETEC strain isolated from piglets with ETEC diarrhea has been completely sequenced. W25K is an O149:K88 serotype strain of ETEC that causes diarrhea in piglets (8).

Genomic DNA was extracted from W25K using a QIAamp DNA minikit (Qiagen), according to the manufacturer’s protocol. One nanogram of genomic DNA was used to generate a library using the Nextera XT kit (Illumina). Micro-Seq Enterprises sequenced the libraries on a MiSeq sequencing system (Illumina). The sequencing generated approximately 4 million read pairs, constituting ~53-fold coverage of the genome. The read pairs were overlapped where possible and trimmed for quality using DNAStar software. A *de novo* assembly of the overlapped and quality-trimmed reads was generated using DNAStar.

The final assembly consists of 153 contigs. The genome size of ETEC W25K is estimated to be 5.6 Mb. The genome sequence was annotated using the RAST genome annotation servers (9). An analysis of the genome showed that ETEC W25K has 599 subsystems, 5,793 coding sequences, and 114 RNAs. Type II and type VI protein secretion systems were identified. Classical ETEC virulence genes, e*ltA* and e*ltB*, the genes encoding the two subunits of heat-labile enterotoxin, were also found.

### Nucleotide sequence accession numbers.

This whole-genome shotgun project has been deposited at DDBJ/EMBL/GenBank under the accession no. JNBX00000000. The version described in this paper is version JNBX01000000.
